# Chromosomal characteristics and distribution of rDNA sequences in the brook trout *Salvelinus fontinalis* (Mitchill, 1814)

**DOI:** 10.1007/s10709-015-9841-6

**Published:** 2015-05-10

**Authors:** A. Śliwińska-Jewsiewicka, M. Kuciński, L. Kirtiklis, S. Dobosz, K. Ocalewicz, Malgorzata Jankun

**Affiliations:** Department of Human Histology and Embryology, Faculty of Medical Sciences, University of Warmia and Mazury in Olsztyn, Olsztyn, Poland; Department of Ichthyology, Faculty of Environmental Sciences, University of Warmia and Mazury in Olsztyn, Olsztyn, Poland; Department of Zoology, Faculty of Biology and Biotechnology, University of Warmia and Mazury in Olsztyn, Olsztyn, Poland; Department of Salmonid Research, Inland Fisheries Institute in Olsztyn, Olsztyn, Poland; Department of Marine Biology and Ecology, Faculty of Oceanography and Geography, University of Gdansk, Gdańsk, Poland

**Keywords:** Salmonid cytogenetics, Chromosome banding, FISH, Karyotype variability, NORs, Telomeres

## Abstract

Brook trout *Salvelinus fontinalis* (Mitchill, 1814) chromosomes have been analyzed using conventional and molecular cytogenetic techniques enabling characteristics and chromosomal location of heterochromatin, nucleolus organizer regions (NORs), ribosomal RNA-encoding genes and telomeric DNA sequences. The C-banding and chromosome digestion with the restriction endonucleases demonstrated distribution and heterogeneity of the heterochromatin in the brook trout genome. DNA sequences of the ribosomal RNA genes, namely the nucleolus-forming 28S (major) and non-nucleolus-forming 5S (minor) rDNAs, were physically mapped using fluorescence in situ hybridization (FISH) and primed in situ labelling. The minor rDNA locus was located on the subtelo-acrocentric chromosome pair No. 9, whereas the major rDNA loci were dispersed on 14 chromosome pairs, showing a considerable inter-individual variation in the number and location. The major and minor rDNA loci were located at different chromosomes. Multichromosomal location (3–6 sites) of the NORs was demonstrated by silver nitrate (AgNO_3_) impregnation. All Ag-positive i.e. active NORs corresponded to the GC-rich blocks of heterochromatin. FISH with telomeric probe showed the presence of the interstitial telomeric site (ITS) adjacent to the NOR/28S rDNA site on the chromosome 11. This ITS was presumably remnant of the chromosome rearrangement(s) leading to the genomic redistribution of the rDNA sequences. Comparative analysis of the cytogenetic data among several related salmonid species confirmed huge variation in the number and the chromosomal location of rRNA gene clusters in the *Salvelinus* genome.

## Introduction

It is considered that the whole-genome duplication (WGD) event dated 88–103 Mya (Macqueen and Johnston 2014) took place in the common ancestor of the Salmonidae and provided the first tetraploid salmonid karyotype composed of about 100 telocentric chromosomes (Phillips and Ráb 2001). To recover disomic segregation duplicated salmonid genome has experienced several chromosomal rearrangements mostly fusions leading to the huge diversification of the karyotypes and adaptive radiations among the extant salmonid species (Phillips and Ráb 2001). Even though, the rediploidization process has not been fully completed yet what is evidenced by the tetrasomic inheritance of some loci (Sakamoto et al. 2000), the formation of the multivalents at meiosis (Oliveira et al. 1995) and the intraspecies chromosome and chromosome arm number variations observed in the representatives of Coregoninae, Salmoninae and Thymallinae subfamilies (Jankun et al. 2007; Caputo et al. 2009; Ocalewicz and Dobosz 2009; Ocalewicz et al. 2013). Apart from the major rearrangements concerning whole chromosomes, translocations, inversions, duplications and deletions of the chromosomal segments have also accompanied evolution of the salmonid karyotypes and resulted in the length and structure polymorphisms of the individual chromosomes.

The genus *Salvelinus* is comprised of three sub-genera: *Salvethymus*, *Baione*, and *Salvelinus* (Nelson 2006). Although, more than 50 species of genus *Salvelinus* has been recognized and described (FishBase 2015) so far only 12 of them have been cytogenetically studied (Arai 2011). In these species, chromosome and chromosome arm numbers range from 76 to 84 and from 98 to 102, respectively and the polymorphisms of the chromosome length and structure are frequently observed (Phillips and Ihssen 1986; Phillips et al. 1989; Fujiwara et al. 1998; Phillips and Ráb 2001). Sex chromosomes have been described in only two *Salvelinus* species (both from subgenus *Baione*); the lake trout (*Salvelinus namaycush*) (Phillips and Ihssen 1985a) and the brook trout (Ocalewicz et al. 2004). Though brook trout was a subject of a number of aquaculture issues (Fischer et al. 2009), genome manipulations (Michalik et al. 2014), population genetics (Danzmann et al. 1998) and phylogenetic studies (Grewe et al. 1990) chromosomal and genomic organization of this salmonid species has been superficially studied to date. Brook trout is the only known salmonid species without described polymorphism regarding diploid chromosome number (2n = 84) and chromosome arm number (FN = 100) (Lee and Wright 1981). On the other hand, brook trout chromosomes exhibit inter-individual variation in the size and position of the GC-rich DNA segments (Phillips and Ihssen 1985b; Phillips and Reed 1996; Ocalewicz et al. 2004) that in fishes are associated with the NORs (Amemiya and Gold 1986; Ráb et al. 1999).

In the present paper we combined conventional and molecular cytogenetic protocols to analyse the brook trout chromosomes in order to explain the extensive variations among the chromatin components of its genome. Combination of silver nitrate (AgNO_3_), chromomycin A_3_ (CMA_3_), 4′,6-diamidino-2-fenylindole (DAPI) staining, C-banding, restriction endonuclease (RE) digestion, fluorescence in situ hybridization (FISH), and primed in situ (PRINS) labelling techniques applied to the brook trout chromosomes enabled chromosomal localization of the major and minor rRNA genes and cytogenetic characteristics of the chromatin overlapping these regions. Extensive polymorphism(s) related to the location of the GC-rich blocks and activity of the NORs observed among studied brook trout individuals has been discussed in the context of enormous dispersion of the 28S rDNA sequences and interstitial location of the telomeric DNA repeats.

## Materials and methods

### Materials

Twenty-two 1-year-old brook trout individuals (10 females, 12 males) were studied cytogenetically. Fish were obtained from an experimental fish farm of the Department of Salmonid Research, Inland Fisheries Institute in Olsztyn, Rutki, Poland. The phenotypic sex was determined in the course of the phase contrast microscope analysis of the gonad tissue squashed with a cover slip.

### Chromosome preparation

Chromosomes were prepared from the pooled cephalic kidney cells by conventional air-drying technique by Jankun et al. (1998). Briefly, fishes were injected with 0.1 % colchicine solution (1 ml/100 g of body weight). After 60 min fishes were sacrificed by overdose of the anesthetic 2-phenoxyethanol (Sigma-Aldrich, USA). Kidney tissue was removed, dissected in 0.075 M KCl and cell suspension free of tissue fragments was hypotonized for 60 min in 0.075 M KCl, prefixed (5 min) and fixed in methanol: acetic acid fixative (3:1), washed twice in fixative, and finally spread onto the microscopic slides.

### Banding techniques

The constitutive heterochromatin blocks were identified using C-banding technique by Sumner (1972) with some modifications. Briefly, slides were treated in 0.1 N hydrochloric acid for 3 min, dipped in saturated Ba(OH)_2_ at 50 °C for 90 s. and incubated in 2 × SSC at 65 °C for 2 h. After washing in distilled water slides were stained with 5 % Giemsa solution for 5 min in pH 6.8. For identification of AT-rich chromatin regions, chromosomes were stained with DAPI. Three drops of antifade solution Vectashield (Vector, Burlingame, USA) containing DAPI (1.5 µg/ml) were dropped onto a slide and covered with a coverslip. Restriction endonuclease (RE) banding was performed by digestion of few hours old spread metaphase chromosomes with *Alu*I, *Dde*I, *Hae*III, *Hin*fI and *Mbo*I (Promega), according to Jankun et al. (2004). Briefly, restriction enzymes suspended in the appropriate buffers were applied in different concentrations to the air-dried cell suspension as shown in Table 1. Then, slides were incubated in a moist chamber at 37 °C, washed with distilled water, and stained with 20 % Giemsa for 17 min, pH 6.8. AgNO_3_ staining of the NORs was performed according to Howell and Black (1980). The CMA_3_ fluorescence banding was performed as described by Sola et al. (1992).

**Table 1:** Restriction enzymes and chromosomes digestion conditions

Name	Recognition sequence	Final concentration (U/µl)	Optimal incubation time at 37 °C (h)
*Alu*I	AG↓CT	0.3	1
*Dde*I	C↓TNAG	0.5	2.3
*Hae*III	GG↓CC	1.5	6
*Hin*fI	G↓ANTC	1.5	4.5
*Mbo*I	↓GATC	1.5	8

### FISH and PRINS

Telomeric DNA repeats were detected by FISH using a telomere Peptide Nucleic Acid (PNA) probe conjugated with FITC (DAKO, Denmark) according to the manufacturer’s protocol. Chromosomal DNA was thermally denatured at 86 °C for 3 min under the coverslip in the presence of the PNA probe. Hybridization took place in the darkness at room temperature for 60 min.

Fluorescence in situ hybridization (FISH) with 28S rDNA probe was performed according to Fujiwara et al. (1998) with slight modification (Kirtiklis et al. 2014). A 28S rDNA probe was obtained via PCR with forward primer F8-28S: 5′-TGAAATACCACTACTCTTATCGTT-3′ and reverse primer R8-28S: 5′-GGATTCTGACTTAGAGGCGTTCAG-3′ (Zardoya and Meyer 1996). A 50 μl reaction mix with 1.0 μM of each of the mentioned above primers, 25 μl of GoTaq Master Mix (Promega, USA), 1 μl of DNA template and nuclease-free water was prepared. PCR product, obtained after 35 cycles of amplification and annealing at 55 °C, was purified using the GeneElute PCR Clean-Up Kit (Sigma, USA), then labeled with Biotin-16-dUTP by nick-translation method (Roche). In situ hybridization with 150 ng of rDNA probe per slide was performed with RNase-pretreated and formamide-denaturated chromosome slides. Post-hybridization wash was performed at 37 °C for 20 min. A detection of rDNA signals were done using avidin-FITC (Roche, Germany).

Primed in situ (PRINS) reaction was carried out according to Ocalewicz et al. (2008). For the chromosomal localization of 5S rDNA sequences, Rhodamine PRINS Labeling kit (Roche, Manheim, Germany) and two primer sequences enabling detection of 5S rDNA: A: (5′-TACGCCCGATCTCGTCCGATC-3′) and B: (5′-CAGGCTGGTATGGCCGTAAGC-3′) were used. After reaction, the slides were transferred to a stop buffer (50 mM EDTA, 50 mM NaCl, pH = 8) for 5 min at 65 °C and washed in Tween 20 in 4 × SSC at room temperature. Chromosomes were counterstained with 25 µl (1.5 µg/ml) DAPI in an antifade solution Vectashield.

### Microscopy and image processing

Metaphase plates were examined using Nikon Optiphot, Nikon 90i (Nikon, Japan) and Zeiss Axio Imager.A1 (Zeiss, Germany) microscopes equipped with epi-fluorescence and the digital cameras. Pictures were acquired using a monochromatic ProgRes MFcool camera (Jenoptic, Germany) controlled by a Lucia software (Laboratory Imaging, Czech Republic). Images of chromosomes after PNA-FISH and PRINS were captured and the electronic processing of the images was performed using Band View/FISH View software (Applied Spectral Imaging, Israel). Post-processing elaboration of all the pictures was made based using CorelDRAW Graphics Suite 11 (Corel Corporation, Canada). The metaphase chromosomes were karyotyped by size and position of the centromere in according to Levan et al. (1964). Metacentrics (m) and submetacentrics (sm) were classified as bi-armed, whereas subtelocentrics (st) and acrocentrics (a) as mono-armed chromosomes.

## Results

**Fig. 1:**
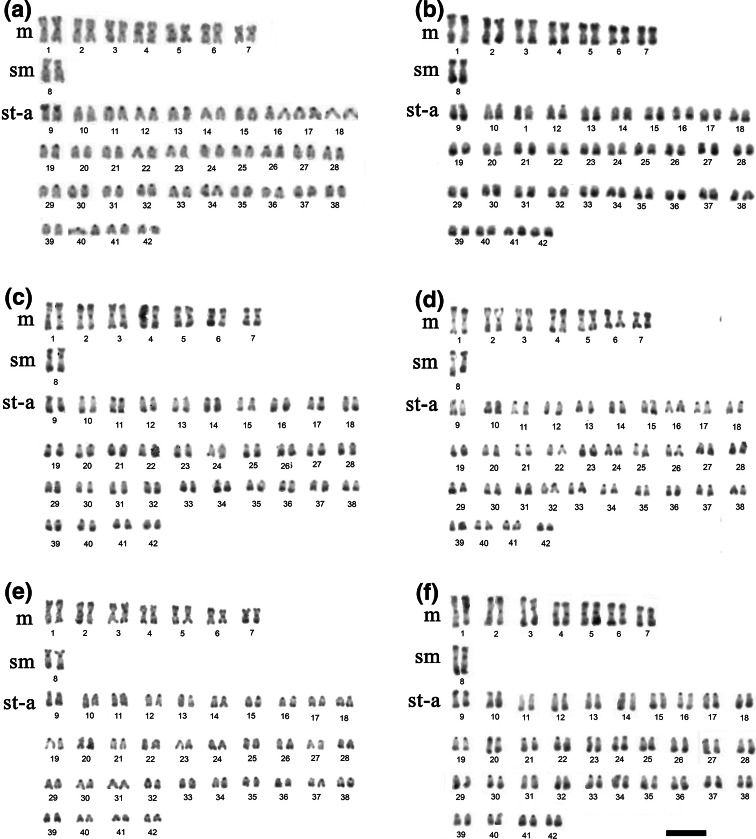
Karyotype of the brook trout (*Salvelinus fontinalis*) arranged from C-banded (**a**), Giemsa-stained after digestion with *Alu*I (**b**), *Dde*I (**c**), *Hae*III (**d**), *Mbo*I (**e**) and *Hin*fI (**f**) restriction endonuclease chromosomes. *Scale bar* = 10 µm

The diploid chromosome number of the brook trout individuals under study was invariably 2n = 84. The karyotype comprised 14 m, two sm and 68 st-a chromosomes (NF = 100) (Fig. 1).

**Fig. 2:**
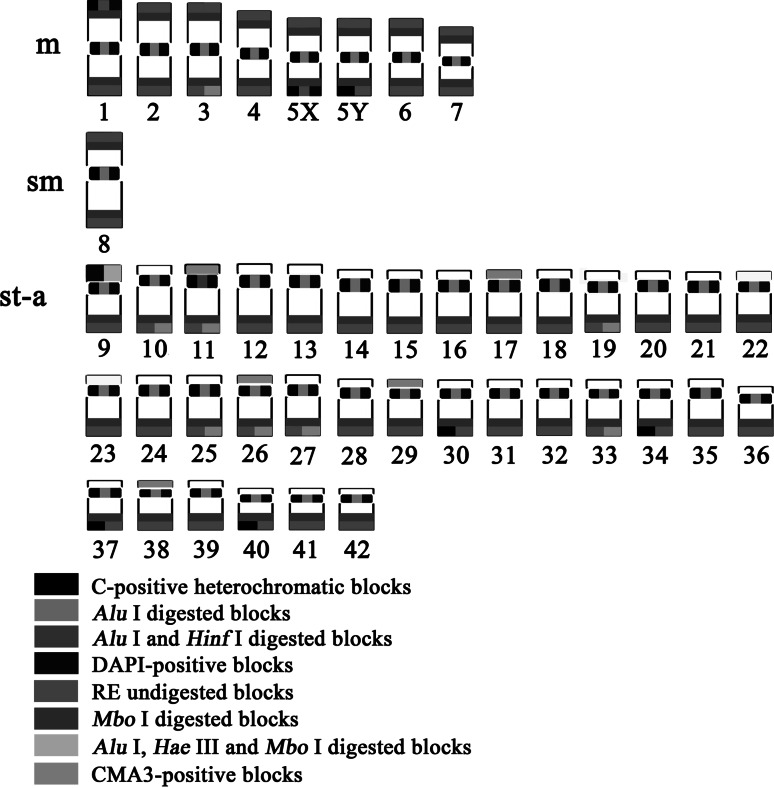
Ideogram representing the distribution of heterochromatin in the chromosomes of the brook trout (*Salvelinus fontinalis*) from Poland

Figure 2 presents patterns of different chromatin types in the karyotype. C-banding revealed the blocks of constitutive heterochromatin located in the pericentromeric regions of all chromosomes. Also telomeric regions of two pairs of m chromosomes (Nos. 1 and 5) and four pairs of a chromosomes (Nos. 30, 34, 37 and 40) were C-positive. Moreover, entire short (*p*) arms of the chromosome pair No. 9 were entirely hetrochromatinized/C-positive (Fig. 1a). Pericentromeric regions of the chromosomes were completely digested by *Alu*I treatment but remained untouched by *Dde*I, *Hae*III and *Mbo*I (Fig. 1b, c, d, e). *Hin*fI digested pericentromeric region of the largest a chromosome pair (No. 11) only (Fig. 1f). Furthermore, entire C-band-positive *p* arms of the chromosome pair No. 9 were digested by *Alu*I, *Hae*III and *Mbo*I (Fig. 1b, d, e). Large blocks of the undigested chromatin were situated at the distal parts of all chromosomes after *Alu*I (Fig. 1b), *Dde*I (Fig. 1c), *Hae*III (Fig. 1d) and *Hin*fI (Fig. 1f) treatments. After *Mbo*I digestion only small telomeric bands remained untouched at all chromosomes (Fig. 1e).

All had pericentromeric bright DAPI signals. DAPI positive telomeric regions were observed in the *q* arms of two m chromosomes No. 5 in the female and only one such chromosome in the male karyotype (Fig. 2). These chromosomes were previously identified as X chromosomes (Phillips et al. 2001; Ocalewicz et al. 2004; Phillips 2013). Moreover, entire *p* arms of st-a chromosomes No. 9 were DAPI-positive and a small telomeric DAPI-positive block on the *p* arm of the largest m chromosome pair (No. 1) was visible (Fig. 2).

**Fig. 3:**
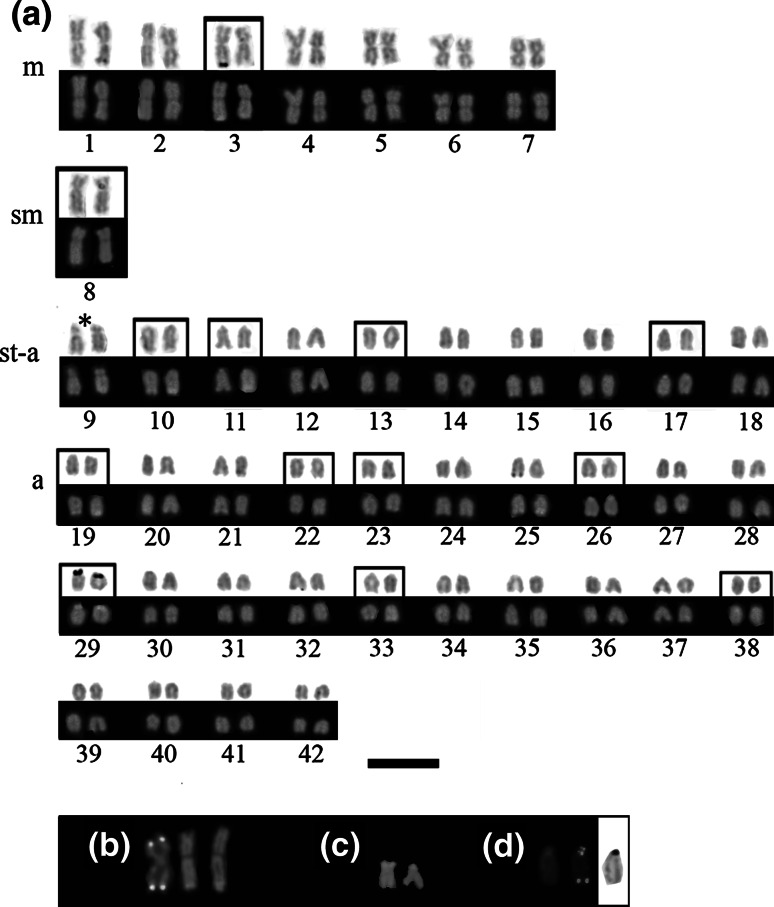
Karyotype of the brook trout (*Salvelinus fontinalis*) arranged from silver-stained (*top row*) and chromomycin A_3_-stained (CMA_3_) (*bottom row*) chromosomes (**a**). Chromosome pairs bearing 28S rDNA are framed, and the chromosome pair with 5S rDNA on the *p* arm is indicated with *asterisk*. Examples of the chromosome pairs Nos. 3 (**b**) and 29 (**c**) showing size polymorphism of GC-rich blocks stained with CMA_3_ and chromosome No. 11 (**d**) displaying internal telomeric site after telomere PNA hybridization (in the *middle*, DAPI on the *left*) which coincided with active NOR (AgNO_3_ staining on the *right*). *Scale bar* = 10 µm

Ag-NORs have been present in 3–6 sites in the karyotype at the chromosome pairs Nos. 3, 10, 11, 26 and 29. One pair of small-sized a chromosomes (No. 29) was AgNO_3_-positive in all studied metaphases (Fig. 3).

Chromomycin A_3_ (CMA_3_)-positive regions were found to be polymorphic in size and number revealing 10–20 chromosomes possessing GC-rich blocks per cell. CMA_3_ signals were found on one m pair (No. 3) and eight or nine st-a chromosome pairs, in some individuals only on one homologue (Fig. 3a). Size polymorphism was recognized in two individuals due to amplification and/or deletions of the CMA_3_-positive region (pairs Nos. 3 and 29) (Fig. 3b, c). Three individuals had two CMA_3_-positive blocks on distal regions of both arms of chromosome No. 3 (Fig. 3b). AgNO_3_-staining performed sequentially after CMA_3_-staining revealed that Ag-positive NORs always occurred only within CMA_3_-positive sites while many small CMA_3_-positive sites never stained by AgNO_3_.

**Fig. 4:**
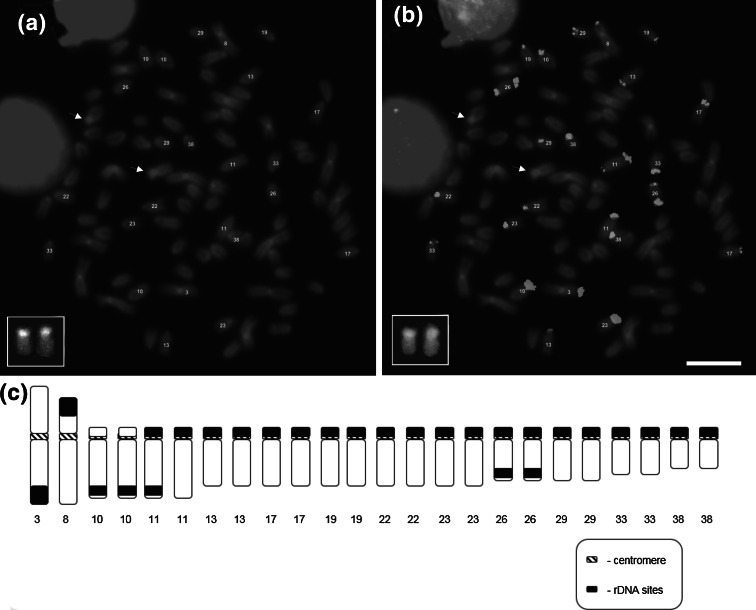
Metaphase plates of *Salvelinus fontinalis* after DAPI staining (**a**), probed with 28S rDNA (**b**) and schematic representation of the chromosomes carrying 28S rDNA sequences (*black*) (**c**); chromosome pair bearing 5S rDNA after DAPI staining is shown in the *inset* in **a** and after PRINS in **b**. *Arrows* point 5S rDNA bearing pair No. 9. *Scale bar* = 10 µm

After FISH with 28S rDNA probe, hybridization signals were mainly observed on one or both arms of 24 chromosomes including one m, one sm and 22 st-a chromosomes (Fig. 4). Fluorescence intensity and number of the chromosomes with fluorescent sites modestly varied intra-individually in the range from 22 to 26 chromosomes (+/−) two signals placed on *p* arms of two st-a chromosomes, signals observed on both arms or only *p* arm of the chromosome No. 11 (Fig. 4). No differences between males and females were detected.

Application of FISH with PNA telomere probe revealed telomeric signals at the ends of the sister chromatids from all chromosomes. Additionally, interstitially located telomeric sequences were observed in the pericentromeric region of the st chromosome No. 11 *p* arm. Sequentially performed PNA-FISH and AgNO_3_-staining exhibited region between terminal and interstitial telomeric signals overlapped by the Ag-positive sites (Fig. 3d).

Primed in situ (PRINS) with primers enabling 5S rDNA sequence amplification revealed that clusters of 5S sequences were located on the whole *p* DAPI-positive arms of the st-a chromosome pair No. 9 (Fig. 4). 5S regions were not associated with NORs.

## Discussion

The karyotypic macrostructure found in *S. fontinalis* (2n = 84, FN = 100) from Polish fish farm confirms the previous analyses of North American (Lee and Wright 1981), Japanese (Ueda and Ojima 1983) and British stocks (Hartley 1991).

Application of the conventional cytogenetic techniques enabled characteristics of the heterochromatin variation in the brook trout. Several types of the C-banded heterochromatin built with predominance of AT base pairs (DAPI-positive), GC base pairs (CMA_3_-positive) and resistant to particular restriction endonucleases grouped in three areas of the brook trout genome including telomeric sites, pericentromeric regions and NORs were distinguished (Figs. 1, 2, 3). Most of the brook trout chromatin is abundant in the DNA sequences recognized by the restriction enzymes used in the present study. Chromatin resistant to all endonucleases was restricted to the telomeric regions. In turn, centromeric regions of the brook trout chromosomes were prone to the digestion of *Alu*I enzyme, only (Fig. 1b). The same properties of centromeric and telomeric chromatin have been reported by Hartley (1991) in the cultured brook trout and the Arctic char (*Salvelinus alpinus*) from England.

About half of the cytogenetically examined salmonid species including e.g. brown trout (*Salmo trutta*), whitefish (*Coregonus lavaretus*), vendace (*Coregonus albula*) and the European grayling (*Thymallus thymallus*) exhibit a multiple NOR-bearing pairs of chromosome (Phillips and Ihssen 1985b; Reed and Phillips 1997; Castro et al. 2001; Jankun et al. 2001, 2003; Frolov and Frolova 2004). Also in fish from genus *Salvelinus* most of the cytogenetically studied species possess multichromosomal location of NORs (Reed and Phillips 1995, 1997; Fujiwara et al. 1998). Mechanisms such as unequal *crossing*-*over* or transposition have been suggested to be responsible for the multichromosomal location and inter-individual variations in size and location of NORs (Zhuo et al. 1995; Reed and Phillips 2000). Reed and Phillips (1995) explained intrapopulation heteromorphism of NORs by translocation rearrangement of rDNA from the distal part of one chromosome to the proximal end of its homologue followed by the recombination between them in the lake trout. Hybridization with telomeric probe, 28S rDNA probe and AgNO_3_ staining performed sequentially in this study showed that, chromosomes No. 11 had interstitial telomeric DNA sequences observed in a margin of the active NOR-related rDNA sequences located on the *p* arm (Fig. 3d). Interestingly, chromosome 11 has also shown another cluster of 28S rDNA sequences located on the *q* arm (Fig. 4). Two other char species, lake trout and the Arctic char exhibit interstitial telomeric DNA repeats that are located close to the GC-rich chromatin (Reed and Phillips 1995; Pomianowski et al. 2012). Although, in fish like in other vertebrates ITSs are usually remnants of the chromosome fusions and less often inversions (Ocalewicz 2013; Ocalewicz et al. 2013) it is not excluded that interstitial insertions of the telomeric DNA repeats may appear in the course of rearrangements responsible for the redistribution of the rDNAs and GC-rich chromatin. However, ITSs are also considered as a fragile sites for the recombination (Ashley and Ward 1993) and potentially may cause redistribution of the DNA sequences, what in turn could result in the polymorphisms of size and location of the GC-rich chromatin in *Salvelinus* species.

In teleost fish species GC-rich CMA_3_ positive sites correspond to the Ag-NOR (e.g. Ráb et al. 1999; Jankun et al. 2003; among others). This is also true for the brook trout studied here showing up to six NORs that are composed of the GC-rich block of chromatin. It has been also observed that not all NORs were active during the last interphase in the studied brook trout (Fig. 3a). This is the manifestation of rRNA gene dosage control. Such system operates in animals to regulate the number of active rRNA genes according to the physiological needs of the cell (McStay and Grummt 2008).

It is very interesting that rDNA sequences that are part of NORs were frequently found on the other than Ag-NOR-carrying brook trout chromosomes (Figs. 3, 4). Supernumerary rDNA loci has been also identified in the brook trout from other populations (Fujiwara et al. 1998; Phillips and Reed 1996) and the lake trout (Reed and Phillips 1995). In *Coregonus fontanae*, apart from the complete 6–10 NORs about 30 rDNA loci located outside NORs were observed. A chromosomal co-localization of the retrotransposon and the rDNAs suggests a retrotransposition of the part of 45S rDNA unit as mechanism responsible for the multiplication and redistribution of the rDNA loci (Symonova et al. 2013). Thus, this may suggest that also in the brook trout most of the supernumerary 28S rDNA hybridization signals were originated from the AT-rich pericentromeric heterochromatin which contain transposable elements. Based on data from several organisms, rRNA gene families seem to evolve according to a combination of the evolutionary processes of birth-and-death and concerted evolution (Rooney and Ward 2005; Eickbush and Eickbush 2007), which could explain the variability observed among related taxa.

In contrast to 28S rDNA sequences, clusters of the non-nucleolus-forming 5S rDNA elements were observed only on one chromosome pair in the brook trout genome. This characteristics is not common for the other chars which rather show multiple locations of the 5S rRNA genes (Phillips et al. 2002). Although, co-localization of the minor and major rDNA loci has been described in several salmonid species (Pendas et al. 1994; Moran et al. 1996) such synteny was excluded in the brook trout.

In conclusion, the present study has shown that a combination of conventional (C-banding, AgNO_3_- and CMA_3_-bandings as well as chromosomal digestion with RE) and molecular cytogenetic techniques (PRINS with primer sequences enabling detection of 5S rDNA and FISH with telomere PNA and 28S rDNA probes) provided original data on the genomic organization of the brook trout chromosomes including distribution and structure of heterochromatin, location of NORs, their activity and physical mapping of NOR-related and the non-nucleolus-forming rDNA sequences.
